# Using drawing and situated learning to teach transitional care to post-graduate residents

**DOI:** 10.1186/s12909-022-03738-4

**Published:** 2022-09-22

**Authors:** Fang-Yih Liaw, Yaw-Wen Chang, Yan-Di Chang, Li-Wen Shih, Po-Fang Tsai

**Affiliations:** 1 Department of Family and Community Medicine, Tri-Service General Hospital, National Defense Medical Center, Taipei, Taiwan; 2 Graduate Institute of Humanities in Medicine, College of Humanities and Social Sciences, Taipei Medical University, No. 250, Wu Shin Street, Taipei City, 110 Taiwan

**Keywords:** Transition of care, Post-graduate medical education, Drawing, Self-reflection, Older adults, Patients with disability

## Abstract

**Background:**

The “draw-and-talk” technique has become popular in medical training, as it can help healthcare practitioners develop empathic understanding of patients and contribute to personal transformation. We adopted this method to make the teaching of transitional care planning more relevant to post-graduate residents undergoing their internal medicine training at a medical center in Taiwan.

**Methods:**

Before the conventional lecture on discharge planning, trainees were invited to draw their “home” and “life as older adults” and share their drawings with others. Subsequently, they were guided to consider whether their home would be livable if they either had a disability or were old. The drawings and narratives were analyzed thematically, and feedback on the session was collected.

**Results:**

Trainees were initially of the opinion that they did not have any role in discharge planning. However, the emphasis on the self-experience of drawing and the thematic use of “home” and “elderly life” led to reflective discussions about post-discharge care. The session provoked constructive self-reflection and meta-cognitive awareness and encouraged residents to actively participate in transition care plans. Response to the draw-and-talk session was overwhelmingly favorable.

**Conclusions:**

Post-graduate residents in Taiwan conventionally do not have much interest or autonomy regarding their patients’ lives outside the hospital. The use of drawing and reflection is a simple and inexpensive method to contextualize discharge planning in participants’ real lives, engage them in actively visualizing the healthcare needs of older adults and patients with disability, and initiate thinking about the impact of discharge preparations, follow-up care, and barriers to care at home. Draw-and-talk might be helpful in improving residents’ knowledge and empathy toward patients preparing for discharge, which is crucial for the quality of transitional care.

**Supplementary Information:**

The online version contains supplementary material available at 10.1186/s12909-022-03738-4.

## Background

Transition of care or transitional care is defined as “a set of actions designed to ensure the coordination and continuity of health care as patients transfer between different sites or levels of care” [1]. It plays an important role in providing high quality care for people with chronic diseases and can also reduce hospital readmissions. After a patient is admitted to the hospital, most interventions begin immediately and are continued for varying time periods after hospital discharge [2]. The Joint Commission, the American Geriatric Society, and the Accreditation Council on Graduate Medical Education (ACGME) have identified care transitions as a core element of patient care and a critically essential component of health professional education [3].

To date, most literature on transitional care has focused on discharges from the hospital to the home. However, details of interventions have not been consistently described. Studies have found variations in the availability of discharge summaries and information such as test results, hospital course, discharge medications, and pending results, which have led to adverse patient outcomes [4, 5]. Few medical residency programs have formal discharge curricula [4, 5]. Teaching on rounds and lectures are the most used method of education for post hospital care transitions [5]; however, these methods lack a “bottom-up” tool to enhance residents’ participation. As a result, residents may not understand the need for or the importance of transitional care.

The most common perception among physician trainees about the cause of problems after discharge is that the discharge was premature or rushed [6]. Currently, Taiwanese PGY residents receive only a few lectures on discharge planning. However, to them, discharge planning lies in the future and is often unpredictable.

**Fig. 1:**
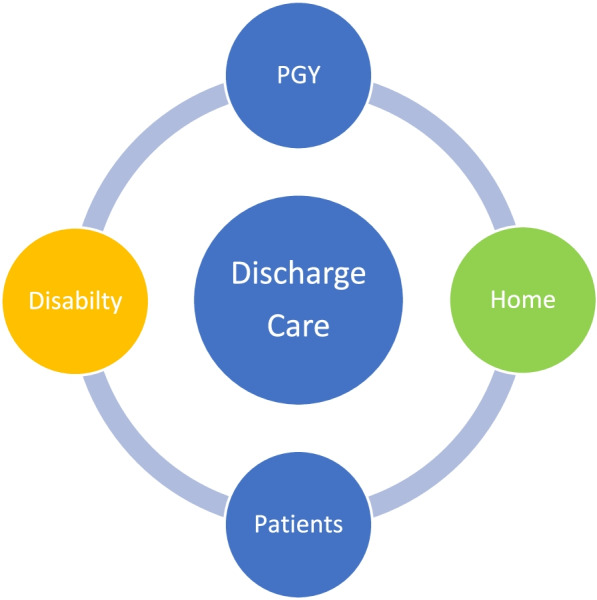
Concept map of the discharge care curriculum

We combined art and situated learning theory (SLT) to teach PGY residents to think about core competencies required for discharged care. Making art can help medical students in family medicine develop empathic understanding of patients [7]. Empathy is a desirable trait for medical professionals [8], and it is important in doctor–patient interactions, as it can increase a patient’s satisfaction [9] and decrease physician burnout [10]. SLT focuses on the relationship between learning and the social situation in which it occurs (Fig. 1 shows the concept map of our curriculum). SLT provides a perspective on learning that is different from the individual-focused views. It is a new way of conceptualizing and studying the learning process, especially in non-classroom settings [11].

Developing a feasible discharge plan that includes support for the transition of care from the hospital to the home setting requires both critical thinking abilities and empathy [6]. Activities of drawing and thinking about home and disability can prime PGY residents to connect more deeply with their patients outside the hospital, following which they can be taught about the importance of the transition of care.

Drawings have long been used to explore conscious and unconscious issues and experiences in social science [12]. The draw-and-talk technique asks participants to draw according to certain prompts and to talk about the meanings expressed through their drawing. The arts and humanities are powerful tools in medical education that have the potential to improve empathy among physicians [13, 14]. Drawings provide a possibility to explore the multiplicity of embodiments, as each drawing may have both a literal and a metaphorical meaning. When combined with oral interviews, it may unfold personal experiences not revealed in the interview alone [15, 16]. As each drawing is unique and personal, drawings provide the chance to access PGY residents’ “situated knowledges” [17] and offer the possibility of understanding their relationship with the setting of the transition of care in the training program. Drawing as it is easy to execute and does not require high-tech or expensive materials (often only a pencil and paper). The benefits of drawing are substantial, including active engagement by adults and visible proof of research findings [18]. To teach the importance of the transition of care, we added a drawing and reflection session before our original curriculum on inpatient medicine for PGY residents.

Teaching discharge planning is important to ensure and sustain the patient’s continuity care by physicians, but current lectures often focus on providing the relevant knowledge. The drawing and sharing would broaden their perspectives about the dilemmas of patients with disability when discharged. This may also prompt them to look for feasible solutions. The purpose of this study was to explore PGYs’ perceptions of the discharged planning and identify opportunities for improvement in education and training programs to improve the PGYs view of transition care.

## Methods

The participants for this study were 20 PGY residents doing their 12-week training in internal medicine at a university hospital that is also a tertiary-care institution between July 2018 and July 2019. Over the year, 20 participants (15 male and 5 female, aged from 27 to 30, and all came from North Taiwan) rotated on the service, with approximately five participants in each session. They were taught about transitional care for patients moving from inpatient to ambulatory care settings, while 8 decided to enter internal medicine, 7 to surgery, and 5 to the other departments after their PGY training year. Although the teaching components included draw-and-talk (60 mins) and lectures (60 mins) within 2 hours, participants were interviewed after the teaching time for at least 30 mins and had a follow-up interview within 1 month. Partially because of the relaxed atmosphere around the draw-and-talk activities, they were not only inclined to, sometimes even well-prepared, take the invitation to participate the interviews, but also fully informed with the options of accept or refuse. We had strong confidences in their willingness to join the study, while there are one or two outliers who had not enough time owning to the emergent calls from their supervisors.

Although three authors with humanities and social sciences background designed the study and another three authors with MD background collecting the data in clinical setting, one faculty FYL, as both researcher and facilitator, led the drawing class and lectures. As far as the aim of the study, the sample specificity, and quality of dialogue are concerned, we believe that the concept of ‘information power’ [19] would be a better guide which satisfied with our simple size (*n* = 20) than the conventional concept of ‘data saturation’ from the grounded theory approach with which the data was analyzed. The rest relevant information about our team reflexivity could be found in Additional file 1: Appendix with COREQ form.

### Initiating reflection

To initiate reflection, we conducted the following activities at the beginning of the course before the lectures:

- Activity 1. Participants were requested to answer the following two questions:
  - Q1. In your opinion, which health care workers belong to the interdisciplinary team for discharge planning? Present the proportion of their involvement with a pie chart.
  - Q2. What do you think about the resident’s role in managing discharge preparations?
- Activity II. Participants were asked to “draw your home” and “draw your vision of your life when you are elderly”. Thereafter, they were asked to share these drawings and their meanings. The facilitator asked two more questions:
  - Q3. Have you ever considered how livable your home would be if you had a disability?
  - Q4. Have you ever wondered how your patients live after they are discharged?

Once all participants had shared their reflections, lectures on discharge planning were given. Topics included the types of patients who require discharge planning, instructions for patients, members of the interdisciplinary team for discharge planning, and the doctor’s role.

- Activity III. At the end of the lecture, participants were asked:
  - Q5. What do you think is the resident’s role in managing discharge preparations?

During the three activities, participants were not only asked to give their answer to five questions, but also led to draw their feelings as pictorial responses to Q1 and Q3 accompanying with their verbal responses. Even if there was no drawing invitation in activity III, most participants could smoothly transfer themselves from a feeling-relaxed and personal-sharing oriented situation back to a lecture-based one for their own reflections. Participants’ oral responses to all the five reflection questions were recorded, and then transcribed for qualitative analysis. All the texts were imported, coded, and exported for in-text quotation through the software MAXQDA version 2018. Although participants’ drawings were examined by all the researchers for developing a consensus on the emerging themes, this analysis was grounded in the participants’ accounts of their drawing. Considering that we need to balance the researcher-instructor’s engaged interpretation and the non-teaching researcher’s detached interpretation at the same time, the team had several exchanges in our analysis stage, both in-person and online. Besides, most themes would not be changed after we considered the verbal accounts because of the ‘picture first, then words’ steps we deployed.

The Institutional Review Board of the Tri-Service General Hospital approved the study (B202005088). Each participant provided informed written consent.

## Results

### Activity I – interview

**Fig. 2:**
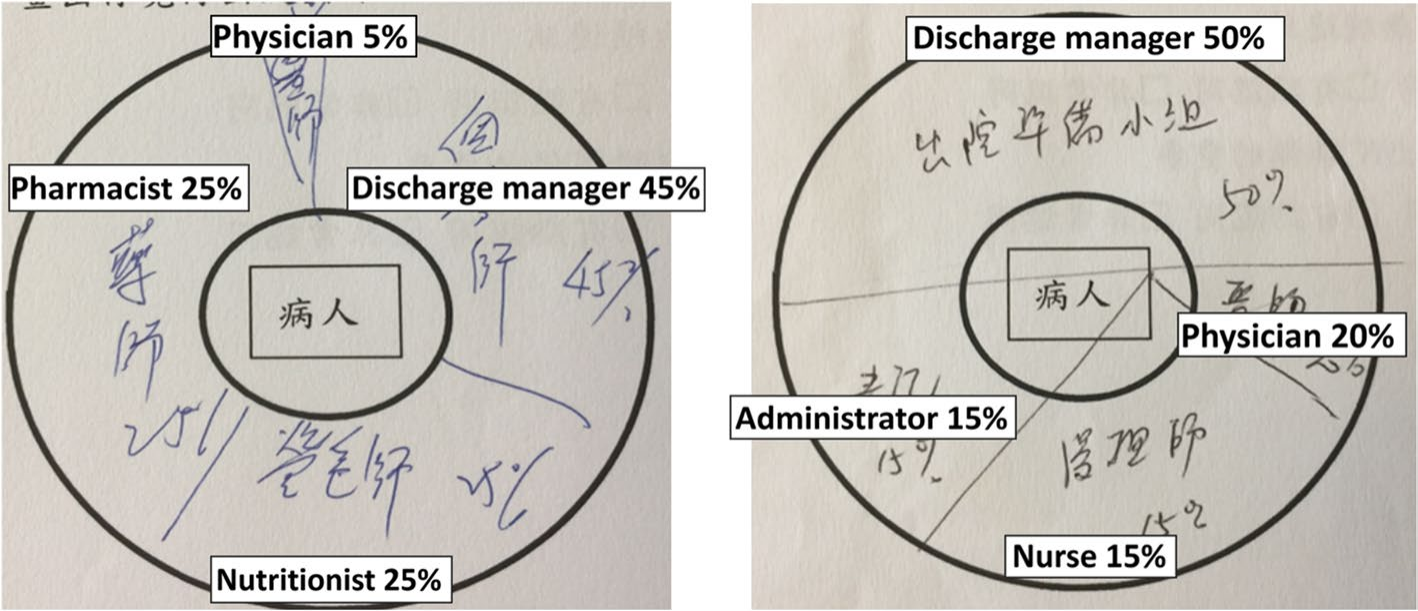
Proportion of importance of the various members of the interdisciplinary discharge planning team members

Figure 2 shows the drawings of two participants for the first question (Q1) before the class.

Participants included the doctor, discharge planning managers, and nurses as members of the interdisciplinary team for discharge planning. Some also included the nutritionist and pharmacist. Although they did include a doctor in the team, they also believed that the doctor has no important role in discharge planning. According to eight participants, the importance of a doctor’s role is less than 20%. A PGY resident’s pie chart indicated that the doctor’s role is only 5%. All participants considered that the discharge planning manager had the most important role (40%).

When the participants were asked, “What do you think about residents’ roles in managing discharge preparing?” (Q2), five participants responded that their primary duty was to treat patients who were admitted and that the post-hospitalization care should be managed by the discharge planning manager. Residents indicated that they would not see the patient after they were discharged; therefore, patients who faced problems after that should visit the outpatient or emergency department, citing the accessibility and convenience of acute care in Taiwan.

### Activity II – personal experience

**Fig. 3:**
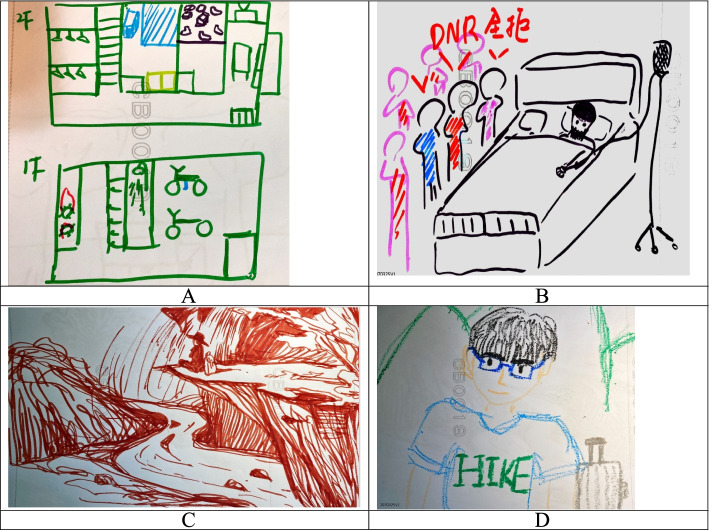
PGY residents’ drawings of their home and older life

Participants’ drawings of their home revealed that 15 lived in apartment buildings with stairs and 5 lived in buildings with elevators. In their drawings, 10 participants conveyed emotions about their home (drawing 5 in Fig. 3A, with the space of his room).

In their drawings of “my life as older adults”, two groups emerged: individuals with disabilities and individuals without disabilities. Examples of these drawings are given in Fig. 3. Most participants (*n* = 16) portrayed themselves as old, with disability, and bedridden (drawing 6 in Fig. 3B, lying on the bed), and half of them (*n* = 10) thought that they would not be able to live in their current homes if they had a disability.

**Fig. 4:**
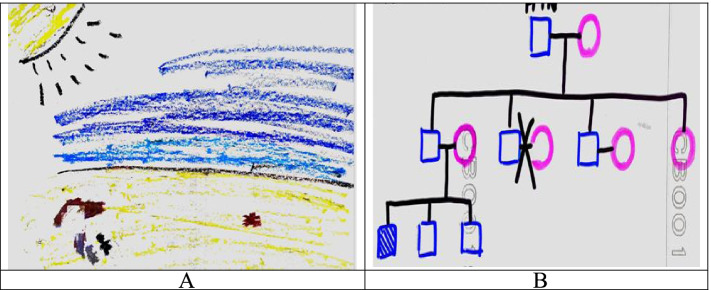
More abstract drawings by PGY residents of their older life and home

In the drawings, various degrees of details and emotions were visible despite the differences in drawing skills. The drawings by participants 9 and 10 (Fig. 3C and D) indicated a good life as older adults, whereas that by PGY14 (Fig. 4A) revealed relatively little knowledge, and that by PGY11 (Fig. 4B) showed symbolic representations.

Even though the drawings produced by the participants were quite unstructured, only four participants drew their life as older adults in health. This contrasts with their relatively good self-reported understanding of illness in older adults.

Overall, we had favorable responses about this pioneer drawing and sharing activity from participants, with 100% of the residents reporting that they found the session useful and learned methods to improve their attitude about the transition of care and discharge procedures.

Some positive comments about the workshop included: “I have not painted for a long time,” “I am very happy in the process of drawing,” and “I didn’t expect that I could have a short period of free drawing time in the midst of our busy clinical training.” The former two comments indicate participant’s interest that might echo the level 1 and 2 (reaction and learning) from Kirkpatrick model, while the latter comment indirectly imply this PGY would be inclined to spend his or her time on free drawing in the future, which slightly corresponding to Kirkpatrick’s level 3 (behavior) [20]. Overall, the participants’ interest in discharge planning was enhanced through the drawing activity.

The reflections of the participants can be classified according to the four phases of approach we modified from trauma-informed care (TIC) [21]: realization, recognition, reflection, and response (Table 1). The TIC concept is an operational framework that can help physicians interact with patients to understand of how trauma impacts the life of an individual seeking services [22]. TIC concept transfers the issue on “what’s wrong with you” to “What happened to you “. Health care worker should know the patient’s life situation and offer effective health care with a healing orientation [22]. In this study, PGYs can reflect on their own imaging trauma experience and how it may influence patient interactions.

**Table 1:** Participants’ phases of reflection

Realization	“[It] lets me recall a lot of things from my childhood.” *(PGY01)* “I am very healthy when I am old and I can do what I like.” *(PGY02)*
Recognition	“When I am old, I will be unhealthy and I will be in bed.” *(PGY13)*
Reflection	“When I am sick, there is no elevator at home, so I will not be able to go out anymore.” *(PGY13)* “There are many places at home that are barrier-free, so life will be harder [when I have a disability].” *(PGY09)* “When I am old, there may be no one around me, perhaps only robots. I don’t know if my savings are enough.” *(PGY07)*
Response	“After imagining yourself getting old through this activity, when you return to the bedside to see your elderly patients now, you will bound to be touched with many feelings. You will now be able to empathize with the many physical pains and sufferings elderly patients have to endure and being hospitalized at this old age, and also understand how inconvenient things can become when they return home.” *(PGY02)* “Before going through this activity, when I take care of patients, the patient’s conditions after discharge did not normally cross my mind. But now I will be especially considerate about the kind of care the patient will need after discharge.” *(PGY07)*

TIC concept included realize the impact of the trauma, recognize the signs of the trauma, responds about trauma into practices, and resist re-traumatization [21]. In our study, the participants were trainees, and we want them to know how important discharge planning is. We collected their response and We found the modified TIC concept is more appropriate from analyzing data. Therefore, we replace the reflect and response to respond and resist.

Participants’ responses indicated that they understood the importance of transition of care and realized the connection between living circumstances and disability. They also recognized some of the possible problems and barriers patients might face when they return home after being discharged. Finally, we believe that they become better prepared to respond and react to transitional care requirements in their daily clinical work.

### Activity III – conclusions

At the end of the lecture, when the participants were again asked about their role in discharge preparations, most participants agreed that they play a significant role during discharge and in transitional care.

“I take care of my patients continuously every day. I know everything about my patients; I know their situation and what they need. I can invite other health professions to help my patient arrange for a customized discharge plan.” *(PGY03).*

“That’s right: I am the person who has the highest level of understanding of my patients during hospitalization. I can help them even after they are discharged.” *(PGY13).*

Through reflection, the lecture achieved the objective of allowing the participants to demonstrate knowledge of the core components of transitional care. The residents identified the curriculum as an effective tool for improving their awareness and attitudes about discharge planning. Participants realized why discharge planning was necessary, recognized what patients needed, reflected upon their actions in daily practice, and responded positively about participating in discharge planning.

## Discussion

This study provided an innovative way of teaching transitions of care to residents. Through these PGYs’ reflections, we have found that they are aware of their important role in discharge planning and willing to play an active role in it.

We use situated learning theory to design the program for PGY to learn the difficulties patients may encounter when returning home. We asked them to draw and reflect. Currently, medical education has increasingly used the arts and humanities as a way of teaching [23]. Drawing can promote higher mental functions supports. It start from simple spontaneous concepts to more complex concepts [24].

The qualitative data were subject to grounded theory approch and guided by theoretical constructs related to modified trauma-informed care. Through modified TIC operational framework, PGYs can connect themselves with the patient and know patient may experience at home. According to Kirkpatrick’s model [20], this curriculum on new teaching and learning methods significantly improved PGYs behavior and transfer into practice in the discharge planning for the patient.

It is recommended that discharging planning curriculum should be considered in educational programs for PGYs.

As Taiwan enters an aging society, medical policy must move with the times. Discharge planning education for physicians is more needed than ever. Previous research is to guide how to perform [25]. This study aims to make them reflect and strengthen their beliefs about discharge planning to care, and furthermore, increase their future practice.

### Bringing PGYs back into rethinking discharge through draw-and-talk

Hospital discharge represents a vulnerable and potentially dangerous transition period [26]. One-third of patients experience an adverse event after discharge, and data from Medicare claims show that approximately one in five patients is readmitted [27]. Transition of care policy statements by the American College of Physicians, Society of General Internal Medicine, Society of Hospital Medicine, and others emphasize the importance of timely and accurate information exchange during transitions of care [28]. Improved communication between inpatient and outpatient care providers allows for smoother transitions of care and helps to ameliorate risk.

In 1993, Taiwan announced a policy to promote discharge planning for all. However, few patients received interdisciplinary discharge planning services initially, as there were no standard procedures at that time. As a result, a high percentage of patients thought that hospitals handled post-discharge long-term care service referrals inadequately. Many physicians still demonstrate an unsatisfactory level of knowledge and behaviors about discharge planning in Taiwan [29]. Improved physician awareness concerning the importance of discharge planning, especially with regards to transitional care, is needed to enhance its implementation [29].

Similarly, in our study, PGY residents initially did not perceive that doctors play an important role in discharge planning. One-third of the PGY residents assumed the involvement of a doctor to be less than 20%. This may be because of the government’s policy and the NHI system, both of which emphasize acute and critical care. Although Taiwan’s healthcare is renowned for its short waiting time and relatively low costs, its weakness is the variable quality of care [30]. It is only recently that efforts to improve long-term care are being put in place [31]. However, the attitudes of senior doctors may influence those of PGY residents. Therefore, it is essential to understand the challenges PGY residents face and counter their stereotypical beliefs.

Through the use of drawing and reflection, this study pushed the PGY residents to reexamine their perceptions about the health care needs of older adults outside the hospital and their needs during transitions of care. We used and modified the approach in trauma-informed care to include four processes: realization, recognition, reflection, and response.

This study analyzed participant responses and used them to develop critical thinking and empathy among the PGY residents by encouraging them to share, reflect, and put themselves in their patients’ shoes when the patients come home.

### Limitations

Art has been used in the teaching of medical humanities for over two decades, for example, paintings have served to enhance observational skills [32], increase the attention span when listening to patients [33], and deepen compassion for suffering [34]. A recent review indicated that drawings have been utilized in virtually all categories of chronic diseases among patients [35]. However, to our knowledge, this might be the first study in Taiwan clinical teaching where drawing has been used as a means to teach transitions of care; traditionally, it is mostly taught through lectures and clinical practice [4].

Drawing their home and life as older adults and answering questions about their patients help connect PGY residents with their patients, as drawings likely bypass issues of social desirability and personal views [35]. Drawings make parts of the self and/or levels of development visible [15]. Artistic images help us access elusive, non-verbalizable aspects of thoughts that might otherwise remain hidden or ignored. Drawing as a research method often entails participants drawing and talking or drawing and writing about the meaning embedded in their drawing [36].

In our study, all PGY residents had different backgrounds and interpretations of “home” and “old age.” Through extended questions, participants learned to think about patients under their care empathetically.

### Implications for educator and researcher

During development of the curriculum, we learned that residents were not addressing discharge planning needs in the hospital course consistently.

The curriculum, designed by us not only serves to standardize the teaching of writing discharge summaries with an emphasis on transition of care but also engages PGY residents in more in-depth communication through drawing. An interactive workshop utilizing real examples from peers led to greater investment and active participation. Residents subjectively felt that the workshop improved their attitude toward utilizing discharge planning for patients in transition. This reflection demonstrated the achievement of the learning objectives.

As facilitators, we found this curriculum easy to implement as it required only a pencil and paper and there was no need for any technological devices. Most importantly, the values imparted through this curriculum can be deeply ingrained in the PGY residents as it is anchored to self-reflection.

Adding a drawing and reflecting section in the teaching of discharge planning ensured that important items were communicated across the transition and led to greater focus on anticipation of ambulatory care needs.

Data collection occurred in a single institutional setting due to limited study resources.. Moreover, the degree of detail in these drawings was insufficient to examine associations between drawing characteristics and other variables. Participants who agreed to respond to the survey may represent a biased sample. Therefore, future studies are recommended to include more participants to establish a larger pool of samples.

At present, there are few published resources on teaching transitions of care using drawing and SLT methodology. Through situated cognition, PGY residents present themselves with the problem and by using drawing and talking, they can communicate and reflect deeply. We believe this curriculum can serve as a useful resource to improve PGY residents’ attitudes and awareness of the importance of documentation in transitions of care.

## Conclusions

The physicians’ attitude about discharge planning is important as adequate attention and empathy toward patients can improve transitional care for patients. In this study, we explored PGY residents’ perspectives through a new curriculum designed to highlight the need for transitional care. Participating residents realized the importance of discharge planning, recognized patients’ needs, reflected on their actions, and adequately responded to participating in future discharge planning.

We enforced the importance of transitional care by using SLT. The action of drawing “home” and “disability” emotionally connected PGY residents to the issues faced by their patients.

Drawing enables individuals to engage in in-depth communication. We believe that the use of drawings is also useful for adults to understand and share memories, thoughts, and feelings that are difficult to put into words. Asking PGY residents to draw their home and life as older adults and then share it with one another may highlight different interpretations of “home” and different perceptions about “old age.” Furthermore, answering extended questions helped participants learn to think about their patients more empathetically. Using the “draw-and-talk” technique as an approach to teaching “transitions of care” leads to a paradigm shift from asking, “What is wrong with this person?” to “What has happened to this person?”

To enhance the implementation of discharge planning in Taiwan, development of a standard lecture on interdisciplinary discharge planning for PGY residents and improved physician realization concerning the importance of discharge planning are needed. Further research is also needed to explore their value to transitional care education. Besides, in our study, the opportunity to draw promoted greater self-awareness and motivation among the PGY residents. The use of art must be further studied to understand its value as a pedagogical tool in teaching core concepts of transitional care and other internal medicine subjects.

Finally, we provide some implications emerging from this study.

First, discharge planning is extremely vital but current pedagogies using lectures or hands-on instruction do not allow learners to understand its importance through examining their own ideas and beliefs about it.

Second, through drawing and reflection, learners could reverse physicians’ ideas about their role in discharge preparation.

Lastly, drawing is a simple and easy activity that can connect people in different fields, time, and spaces, and is suitable for teaching discharge care planning.

## Supplementary Information

**Additional file 1: Appendix.** Consolidated criteria for reporting qualitative studies (COREQ): 32-item checklist.

## Data Availability

The datasets and/or analyzed during the current study available from the corresponding author on reasonable request due to privacy concerns.

## Abbreviations

PGY: Post-graduate Year
SLT: Situated Learning Theory
NHI: National Health Insurance
