# Supplementary material for: Using drawing and situated learning to teach transitional care to post-graduate residents
Source: BMC Med Educ. 2022 Sep 22;22:687. doi: 10.1186/s12909-022-03738-4 (PMC9494879; doi:10.1186/s12909-022-03738-4)
Supplement: Supplementary file 1 — Additional file 1: Appendix. Consolidated criteria for reporting qualitative studies (COREQ): 32-item checklist. [file 12909_2022_3738_MOESM1_ESM.docx]

Appendix:

Consolidated criteria for reporting qualitative studies (COREQ): 32-item checklist

| No. Item | Guide question | Reported on Page# |
| --- | --- | --- |
| **Domain 1: Research team and reflexivity** | | |
| *Personal Characteristics* | | |
| 1. Interviewer/facilitator | Which author/s conducted the interview or focus group? | Method section, page 10 |
| 2. Credentials | What were the researcher’s credentials? E.g. PhD, MD | Method section, page 10.  2 MD, 2 PhD, and 1 MD/PhD. |
| 3. Occupation | What was their occupation at the time of the study? | Method section, page 10.  2 clinical educator, 2 academic faculty, and 1 mixed researcher. |
| 4. Gender | Was the researcher male or female? | N/A. 3 female and 2 male. |
| 5. Experience and training | What experience or training did the  researcher have? | Method section, page 10.  2 clinical educator in family medicine (1 MA in medical humanities), 2 academic faculty (sociology and STS), and 1 mixed (family medicine and anthropology) |
| *Relationship with participants* | | |
| 6. Relationship established | Was a relationship established prior to study commencement? | Method section, page 10-11. Yes, the first author’s MA in Medical Humanities as the key link in our team. |
| 7. Participant knowledge of the interviewer | What did the participants know about the researcher? e.g. personal goals, reasons for doing the research | Method section, page 10-11. They know the researcher-instructor during their PGY training, and have high willingness to participate. |
| 8. Interviewer characteristics | What characteristics were reported about the inter viewer/facilitator? e.g. Bias, assumptions, reasons and interests in the research topic | Method section, page 10. The first author as a mixed (interviewer/ facilitator). |
| **Domain 2: study design** | | |
| *Theoretical framework* | | |
| 9. Methodological orientation and Theory | What methodological orientation was stated to underpin the study? e.g. grounded theory, discourse analysis, ethnography, phenomenology, content analysis | Methods section, page 12-13. We deploy a participant action research in educational context with three stages (activity I, II, and III). In this regard, our analysis was oriented to thematic analysis based on grounded theory plus iconographical analysis. |
| *Participant selection* | | |
| 10. Sampling | How were participants selected? e.g.  purposive, convenience, consecutive,  snowball | Methods section, page 9-10. We deploy a convenience sampling. |
| 11. Method of approach | How were participants approached? e.g. face-to-face, telephone, mail, email | Method section, page 10-11. The participants were approached through face-to-face way. |
| 12. Sample size | How many participants were in the study? | Methods section, page 9-10. There were 20 participants. |
| 13. Non-participation | How many people refused to participate or dropped out? Reasons? | Methods section, page 9-10. There was no refuse or drop out because of the smooth and relaxed atmosphere. |
| *Setting* | | |
| 14. Setting of data collection | Where was the data collected? e.g. home, clinic, workplace | Methods section, page 9-10. Data was collect at workplace during PGY training class. |
| 15. Presence of non-participants | Was anyone else present besides the  participants and researchers? | Methods section, page 9-10. There was neither non-participants, nor non-researcher present. |
| 16. Description of sample | What are the important characteristics of the sample? e.g. demographic data, date | Methods section, page 10. We provide the descriptions of gender, age, dwell location, and professional path in the future. |
| *Data collection* | | |
| 17. Interview guide | Were questions, prompts, guides provided by the authors? Was it pilot tested? | Methods section, page 11-12. We deploy 5 reflection questions and 2 invitations of drawing, but no pilot test. |
| 18. Repeat interviews | Were repeat interviews carried out? If yes, how many? | N/A |
| 19. Audio/visual recording | Did the research use audio or visual recording to collect the data? | Methods section, page 12-13. We deploy both audio recording and pictorial drawing to collect data. |
| 20. Field notes | Were field notes made during and/or after the interview or focus group? | N/A |
| 21. Duration | What was the duration of the interviews or focus group? | Methods section, page 10. Draw-and-talk cost around 60 mins, and interview cost 0.5 hour. |
| 22. Data saturation | Was data saturation discussed? | Methods section, page 11.With 4-5 rounds conducted, we believe the data achieve its saturation. |
| 23. Transcripts returned | Were transcripts returned to participants for comment and/or correction? | N/A |
| **Domain 3: analysis and findings** | | |
| *Data analysis* | | |
| 24. Number of data coders | How many data coders coded the data? | Methods section, page 13. Five authors had discussion on the emerging themes, yet the first author coded the data. |
| 25. Description of the coding tree | Did authors provide a description of the coding tree? | N/A |
| 26. Derivation of themes | Were themes identified in advance or derived from the data? | Methods section, page 13. Although emerging themes came first from participant’s drawings, they were derived from the data, both pictorial and verbal accounts. |
| 27. Software | What software, if applicable, was used to manage the data? | MAXQDA 2018 |
| 28. Participant checking | Did participants provide feedback on the findings? | N/A |
| *Reporting* | | |
| 29. Quotations presented | Were participant quotations presented to illustrate the themes/findings? Was each quotation identified? e.g. participant number | Results section, page 13-18. We deploy not only participant’s drawings, but also their quotations to illustrate the themes. Besides, all the quotations were identified with participant number e.g. *(PGY03).* |
| 30. Data and findings consistent | Was there consistency between the data presented and the findings? | Results section, page 13-18. Yes, there was consistency between the data and the themes. |
| 31. Clarity of major themes | Were major themes clearly presented in the findings? | Results section, page 13-18. Yes, the major themes were presented in the Results section. |
| 32. Clarity of minor themes | Is there a description of diverse cases or discussion of minor themes? | N/A |
